# Stolonifera from shallow waters in the north-western Pacific: a description of a new genus and two new species within the Arulidae (Anthozoa, Octocorallia)

**DOI:** 10.3897/zookeys.790.28875

**Published:** 2018-10-15

**Authors:** Yee Wah Lau, Frank Robert Stokvis, Leendert Pieter van Ofwegen, James Davis Reimer

**Affiliations:** 1 Molecular Invertebrate Systematics and Ecology Laboratory, Graduate School of Engineering and Science, University of the Ryukyus, 1 Senbaru, Nishihara, Okinawa 903-0123, Japan; 2 Naturalis Biodiversity Center, Darwinweg 2, 2333 CR Leiden, The Netherlands; 3 Tropical Biosphere Research Center, University of the Ryukyus, 1 Senbaru, Nishihara, Okinawa 903-0123, Japan

**Keywords:** Arulidae, COI, molecular phylogeny, mtMutS, north-western Pacific, octocoral, 28S rDNA, Stolonifera, taxonomy

## Abstract

A new genus and two new species of stoloniferous octocorals (Alcyonacea) within the family Arulidae are described based on specimens collected from Okinawa (Japan), Palau and Dongsha Atoll (Taiwan). *Hana***gen. n**. is erected within Arulidae. *Hanahanagasa***sp. n.** is characterised by large spindle-like table-radiates and *Hanahanataba***sp. n.** is characterised by having ornamented rods. The distinction of these new taxa is also supported by molecular phylogenetic analyses. The support values resulting from maximum likelihood and Bayesian inference analyses for the genus *Hana* and new species *H.hanagasa* and *H.hanataba* are 82/1.0, 97/1.0 and 61/0.98, respectively. *Hanahanagasa***sp. n.** and *Hanahanataba***sp. n.** are the first arulid records for Okinawa, Palau, and Dongsha Atoll, and represent species of the second genus within the family Arulidae.

## Introduction

Stolonifera is a subordinal group within Octocorallia, consisting of octocoral families that have been grouped together based mainly on the character of having polyps that arise separately from an encrusting horizontal, branching, ribbon-like stolon, or with polyps arising from broad, encrusting membranes. Stoloniferans are therefore morphologically different from other octocorals, which have their polyps embedded within common coenenchymal tissue. Like soft corals, stoloniferan octocorals are found in various marine ecosystems, such as coral reefs in shallow tropical and temperate seas (Fabricius and Alderslade 2001; Daly et al. 2007, McFadden and Ofwegen 2012). Relative to some other octocoral groups (soft corals and gorgonians) little is known about stoloniferan octocorals, and this is especially true concerning molecular studies. Most Stolonifera studies involve the formal description of new species based on historical alpha-taxonomy methodology (Ofwegen et al. 2006, Alderslade and McFadden 2007, Williams 2013, Churashima Foundation and Biological Institute on Kuroshio 2016). The most comprehensive study on stoloniferan phylogenetic relationships to date was conducted by McFadden and Ofwegen (2012). Their results demonstrated that there is still much work to be done for this taxonomic group and confirmed the polyphyletic distribution of Stolonifera within Alcyonacea.

Until 2012, there were six families of Alcyonacea considered to belong to the Stolonifera; Acrossotidae Bourne, 1914, Coelogorgiidae Bourne, 1900, Cornulariidae Dana, 1846, Clavulariidae Hickson, 1894, Pseudogorgiidae Utinomi & Harada, 1973, and Tubiporidae Ehrenberg, 1828. Of these, the family Clavulariidae is the most speciose and most studied, comprising 27 genera and over 60 species (Cordeiro et al. 2018). The other five families are all either monospecific or monogeneric; having no more than a few described species. A seventh monotypic family, Arulidae, was erected in 2012 (McFadden and Ofwegen 2012), describing the single genus *Arula* and the single species *Arulapetunia*, collected from subtropical South African waters. Arulidae is characterised by having polyps with tentacles that are fused together proximally, forming an expanded and broad circular oral membrane. Arulidae also has ‘table-radiate’ sclerites that are altar-like shaped, which had never been recorded before in any other octocoral species. The known distribution of *Arulapetunia* is from the east coast of South Africa from Tanskei to northern Natal, and there are additional photographic records of similar species or relatives from Bali, Indonesia (McFadden and Ofwegen 2012), Oman (Weinberg 2017) and Sabah, Malaysia (Lau pers. obs.).

Recent observations and collections in the north-western Pacific have revealed a similar abundance of stoloniferous octocoral species in coral reefs that are either unrecorded or even undescribed (Churashima Foundation and Biological Institute on Kuroshio 2016). Many stoloniferan octocorals have small and inconspicuous polyps that are usually only ~2–3 mm in diameter and are often overlooked. These octocorals could potentially fill up important knowledge gaps concerning phylogenetic relationships within the subordinal group Stolonifera, as this group is polyphyletic within the Octocorallia. Thus, there is a need to investigate and identify Stolonifera in this region. Here, we describe a new genus and two new species within the family Arulidae from recent collections in Okinawa (Japan), Palau, and Dongsha Atoll (Taiwan), which are situated in the north-western Pacific.

## Methods

### Specimen collection

**Figure 1.**
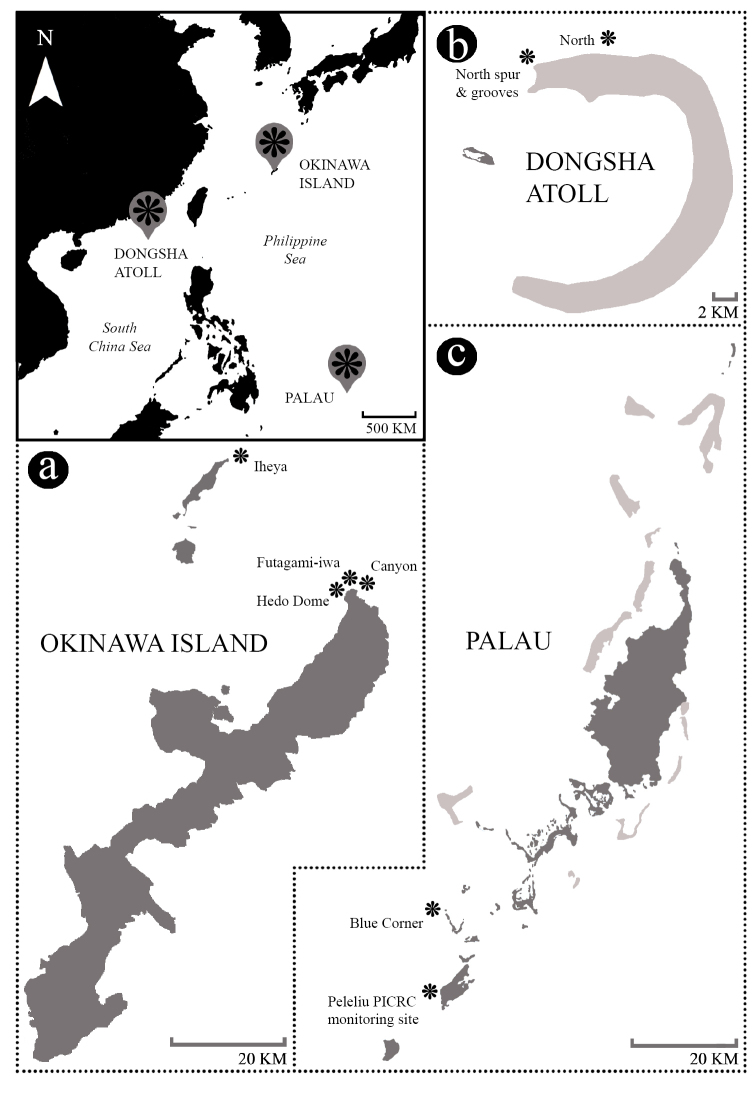
Map of sampling sites at three locations in the north western Pacific; **a** Okinawa Island (Japan) **b** Dongsha Atoll (Taiwan); and **c** Palau.

A total of 16 arulid specimens were collected, at Okinawa Island, Japan (n=12) from June to August 2017, at Palau (n=2) from December 2017 to January 2018, and at Dongsha Atoll, Taiwan (n=2) from April to May 2018. All specimens were collected at depths between 5–30 m by means of SCUBA. Material was preserved in 99% ethanol. In total eight localities were visited for sampling, Okinawa Island (n = 4), Palau (n = 2), and Dongsha Atoll (n = 2) (Figure 1). An overview with collection data of the specimens is presented in Table 1. Vouchers and type material have been deposited at the National Museum of Nature and Science, Tokyo, Japan.

**Table 1.** Information on voucher specimens and GenBank accession numbers of stoloniferan octocoral taxa and reference taxa used in phylogenetic analyses in this study. Collection numbers: NTM=Museum and Art Gallery of the Northern Territory; RMNH=Naturalis Biodiversity Center; USNM=National Museum of Natural History (Smithsonian Inst.). Voucher numbers: OKA=Okinawa Island; ROR=Palau; DSX=Dongsha Atoll. Genbank accession number (AN), n.a.=not available.

Family	Genus/species	Specimen voucher	Location	GPS (DDM)	Genbank AN		
					COI	mtMutS	28S rDNA
Arulidae	* Arula petunia *	RMNH Coel. 40188	South Africa	Mcfadden and Ofwegen 2012	JX203827	JX203773	JX203670
		USNM 1178392	South Africa	Mcfadden and Ofwegen 2012	JX203828	JX203774	JX203671
	*Hanahanagasa* gen. n., sp. n.	OKA170629-01	Iheya – Iheya Island, Okinawa Island, Japan	27°5.710’N, 128°1.828’E	MH845559	n.a.	MH844382
		OKA170711-06	Hedo Dome – Cape Hedo, Okinawa Island, Japan	26°51.125’N, 128°15.027’E	MH845552	n.a.	n.a.
		OKA170711-07	Hedo Dome – Cape Hedo, Okinawa Island, Japan	26°51.125’N, 128°15.027’E	n.a.	n.a.	n.a.
		OKA170711-08	Hedo Dome – Cape Hedo, Okinawa Island, Japan	26°51.125’N, 128°15.027’E	MH845553	n.a.	n.a.
		OKA170711-10	Hedo Dome – Cape Hedo, Okinawa Island, Japan	26°51.125’N, 128°15.027’E	MH845554	MH845544	n.a.
		OKA170711-16	Hedo Dome – Cape Hedo, Okinawa Island, Japan	26°51.125’N, 128°15.027’E	n.a.	n.a.	n.a.
		OKA170711-15	Canyon – Cape Hedo, Okinawa Island, Japan	26°52.326’N, 128°15.995’E	MH845555	MH845545	n.a.
		OKA170711-17	Canyon – Cape Hedo, Okinawa Island, Japan	26°52.326’N, 128°15.995’E	n.a.	MH845546	n.a.
		OKA170711-20	Hedo Dome – Cape Hedo, Okinawa Island, Japan	26°51.125’N, 128°15.027’E	MH845556	MH845547	MH844383
		OKA170818-03	Futagami-iwa – Cape Hedo, Okinawa Island, Japan	26°52.177’N, 128°14.847’E	MH845557	n.a.	MH844384
		OKA170818-05	Futagami-iwa – Cape Hedo, Okinawa Island, Japan	26°52.177’N, 128°14.847’E	n.a.	n.a.	n.a.
		OKA170818-11	Canyon – Cape Hedo, Okinawa Island, Japan	26°52.326’N, 128°15.995’E	MH845558	n.a.	n.a.
		ROR171225-01	Blue Corner – Ngemelis Island, Palau	7°8.400’N, 134°13.200’E	MH845550	n.a.	MH844386
		ROR171226-03	Peleliu PICRC monitoring site – Peleliu, Palau	7°0.400’N, 134°13.060’E	MH845551	MH845543	MH844387
		DSX180420-1-01	North spur & grooves – Dongsha Atoll, Taiwan	20°46.291’N, 116°46.057’E	MH845548	n.a.	n.a.
		DSX180424-3-15	North – Dongsha Atoll, Taiwan	20°46.677’N, 116°50.090’E	MH845549	MH845542	MH844385
Clavulariidae	*Paratelesto* sp.	RMNH Coel. 40019	Mcfadden & Ofwegen, 2012	Mcfadden and Ofwegen 2012	GQ342411	GQ342489	JX203693
	*Rhodelinda* sp.	NTM C12792	Mcfadden & Ofwegen, 2012	Mcfadden and Ofwegen 2012	JX203845	n.a.	JX203695

### Morphological study

Sclerites were isolated by dissolving entire polyps and stolons in 4% hypochlorite (household bleach). Sclerites were rinsed at least seven times with de-ionised water, dried, and initially studied by embedding the sclerites in Euparal on glass slides. In addition, for more detailed morphological studies, sclerites were mounted on scanning electron microscope (SEM) stubs and coated with Pd/Au for imaging on a JEOL JSM6490LV SEM operated at high vacuum at 15kV.

### DNA extraction, amplification, and sequencing

DNA was extracted from polyps, using a DNeasy Blood and Tissue kit (Qiagen, Tokyo). PCR was performed for two mitochondrial markers, cytochrome c oxidase subunit I (COI) and the MSH homologue mtMutS. The nuclear ribosomal marker, 28S rDNA, was amplified as well. The ~900 bp fragment of COI was amplified using the primers COII8068xF 5’-CCATAACAGGACTAGCAGCATC-3’ and COIOCTr 5’-TCATAGCATAGACCATACC-3’ (McFadden et al. 2011) in 20 µL PCR reaction mixes, containing 10 µL of HotStarTaq master mix, 7 µL of RNase-free water, 1 µL of each primer, and 1 µL DNA template. The amplification protocol consisted of 3 min of initial denaturation at 95 °C followed by 39 cycles of 10 sec at 95 °C, annealing at 58 °C for 1 min, extension at 72 °C for 1 min and a final extension at 72 °C for 5 min. An ~800 bp fragment of mtMutS was amplified using the primers ND42599F 5’-GCCATTATGGTTAACTATTAC-3‘ (France and Hoover 2002), and MUT3458R 5’-TSGAGCAAAAGCCACTCC-3’ (Sánchez et al. 2003). Reactions for mtMutS were carried out in 25 µL reaction mixes, containing 0.25 µL Taq DNA polymerase, 0.5 µL dNTP’s, 1 µL BSA, 2.5 µL Coral Load Buffer, 1 µL MgCl_2_, 16.8 µL RNase-free water, 1 µL of each primer, and 1 µL DNA template. PCR conditions were similar to COI, with the exception of the 48 °C annealing temperature. The ~900 bp 28S rDNA fragment was amplified using the primers 28S-Far 5’-CACGAGACCGATAGCGAACAAGTA-3’ and 28S-Rar 5’-TCATTTCGACCCTAAGACCTC-3’ (McFadden and Ofwegen 2013). An annealing temperature of 50 °C was used for 28S. Amplified PCR fragments were purified using the standard ExoSAP protocol and sent for bidirectional sequencing on an ABI 3730XL (Fasmac, Kanagawa, Japan). Sequences were assembled and edited using Geneious R11 (Kearse et al. 2012) and BioEdit (Hall 1999). COI and mtMutS were checked for stop-codons in AliView (Larsson 2014).

### Molecular phylogenetic analyses

Multiple sequence alignments were performed using MAFFT 7 (Katoh and Standley 2013) under default parameters. Consensus sequences for each marker were aligned to a reference dataset of four octocoral taxa (McFadden and Ofwegen 2012), including two stoloniferan specimens of the family Arulidae, *Arulapetunia*. The nearest sister taxa, *Paratelesto* sp. and *Rhodelinda* sp., were used as outgroup taxa in the alignments. Subsequently, alignments of 909 bp for COI, 714 bp for mtMutS and 825 bp for 28S rDNA were obtained. Each dataset was separately run for maximum likelihood (ML) analyses, to check for contamination [Suppl. material 1]. All new sequences generated in this study were deposited in GenBank (Table 1). Maximum likelihood and Bayesian inference were performed on the Naturalis OpenStack computing cloud using PhylOstack (Doorenweerd 2016). Alignments of different markers were concatenated using SequenceMatrix 1.8 (Gaurav et al. 2011), resulting in a 2448 bp dataset of 17 taxa. Maximum likelihood analyses were run with RAxML 8 (Stamatakis 2014) using the GTRCAT model. The best maximum likelihood tree was calculated using the –D parameter. A multi-parametric bootstrap search was performed, which automatically stopped based on the extended majority rule criterion. The resulting RaxML bootstrap tree was analysed with RogueNaRok (Aberer et al. 2013). The Bayesian inference was performed with ExaBayes 1.5 (Aberer et al. 2014) using the GTR substitution model. Four independent runs with each four Monte Carlo Markov Chains were run for 1,000,000 generations during which convergence, with a standard deviation of split frequencies <2%, had been reached. The effective sample size was confirmed using Tracer 1.6.0 (Rambaut et al. 2014). Bootstrap supports and posterior probabilities were depicted on the branches of the best maximum likelihood tree using P4 (Foster 2004). The resulting tree was visualized in FigTree 1.4.2 (Rambout 2014). Additionally, average distance estimations within species and within genera were computed using MEGA7 (Kumar et al. 2016) by analysing pairwise measures of genetic distances (uncorrected *P*) among sequences.

## Systematics

### Class Anthozoa Ehrenberg, 1831

#### Subclass Octocorallia Haeckel, 1866

##### Order Alcyonacea Lamouroux, 1812

###### Family Arulidae

Taxon classificationAnimaliaAlcyonaceaArulidae

McFadden & Ofwegen, 2012

####### Type genus.

*Arula* McFadden & Ofwegen, 2012

####### Diagnosis

(after McFadden and Ofwegen 2012). Alcyonacea with polyps that have tentacles that are fused proximally into a broad, circular oral membrane. Sclerites in the form of table-radiates.

###### Genus Hana gen. n.

Taxon classificationAnimaliaAlcyonaceaArulidae

http://zoobank.org/E1625D14-C9E7-4106-8B5E-1518D6C9F81B

####### Type species.

*Hanahanagasa*, sp. n., by original designation.

####### Diagnosis.

Colony with polyps connected through flat and thin ribbon-like stolons. Anthocodiae (retractile portion of polyp) retract into cylindrical to clavate calyces. Tentacles are fused proximally, forming a broad, circular oral membrane. The oral membrane has eight deep furrows, which run from the intertentacular margin to the mouth of the polyp, giving it a plump appearance. Sclerites of anthocodia are rods. Sclerites of calyx are 6-radiates and table-radiates. The main difference between *Hana* and *Arula* is in sclerites found in the type species *Hanahanagasa* sp. n. and *Arulapetunia* in the stolon. Sclerites of the stolon are fused sheets that form a flattened network of table-radiates in *H.hanagasa*, while in *A.petunia* they are similar to the separate table-radiates found in the calyx. Additionally, there is a difference in sizes of the table-radiates, being longer in *H.hanagasa* than in *A.petunia*. Sclerites colourless. Zooxanthellate.

####### Etymology.

From the Japanese language ‘hana’ (花), meaning flower; denoting the shape of the polyps, which resemble flowers. Gender: feminine.

###### Hana hanagasa sp. n.

Taxon classificationAnimaliaAlcyonaceaArulidae

http://zoobank.org/698530D5-AD0B-4406-BB66-49547647E629

Figures 2a , 3

####### Material examined.

All specimens are from Okinawa Island, Okinawa, Japan. Holotype: OKA170711-15, Canyon, Cape Hedo (26°52.326'N, 128°15.995'E), 17 m depth, coll. YW Lau, 11 July 2017 (MH845555; MH845545). Paratype 1: OKA170629-01, Iheya, Iheya Island (27°5.710'N, 128° 1.828'E), coll. R Janssen, 29 June 2017 (MH845559; MH844382). Paratype 2: OKA170711-06, Hedo Dome, Cape Hedo (26°51.125'N, 128°15.027'E), 6 m depth, coll. YW Lau, 11 July 2017 (MH845552). Paratype 3: OKA170711-07, Hedo Dome, Cape Hedo (26°51.125'N, 128°15.027'E), 6 m depth, coll. YW Lau, 11 July 2017. Paratype 4: OKA170711-08, Hedo Dome, Cape Hedo (26°51.125'N, 128°15.027'E), 10 m depth, coll. YW Lau, 11 July 2017 (MH845553). Paratype 5: OKA170711-10, Hedo Dome, Cape Hedo (26°51.125'N, 128°15.027'E), 11 m depth, coll. YW Lau, 11 July 2017 (MH845554; MH845544). Paratype 6: OKA170711-16, Hedo Dome, Cape Hedo (26°51.125'N, 128°15.027'E), 7 m depth, coll. FR Stokvis, 11 July 2017. Paratype 7: OKA170711-17, Canyon, Cape Hedo (26°52.326'N, 128°15.995'E), 16 m depth, coll. FR Stokvis, 11 July 2017 (MH845546). Paratype 8: OKA170711-20, Hedo Dome, Cape Hedo (26°51.125'N, 128°15.027'E), coll. JD Reimer, 11 July 2017 (MH845556; MH845547; MH844383). Paratype 9: OKA170818-11, Canyon, Cape Hedo (26°52.326'N, 128°15.995'E), collected by JD Reimer, 18 August 2017 (MH845558). Paratype 10: OKA170818-03, Futagami-iwa, Cape Hedo (26°52.177'N, 128°14.847'E), 22 m depth, coll. YW Lau, 18 August 2017 (MH845557; MH844384). Paratype 11: OKA170818-05, Futagami-iwa, Cape Hedo (26°52.177'N, 128°14.847'E), 11 m depth, coll. JD Reimer, 18 August 2017.

####### Description.

The colony consists of numerous small polyps (~50) growing on hard coral rock. Polyps are spaced apart irregularly (0.3–2.5 mm), connected by stolons that are 0.5 mm in diameter and flat thin ribbon-like in cross-section. Polyps have anthocodia fully retracted into calyces of 2.5–3 mm tall and up to 1.0 mm diameter at the widest point; calyces are slightly club-shaped or barrel shaped, wider near the distal end than at the proximal point of attachment to the stolon.

**Figure 2.**
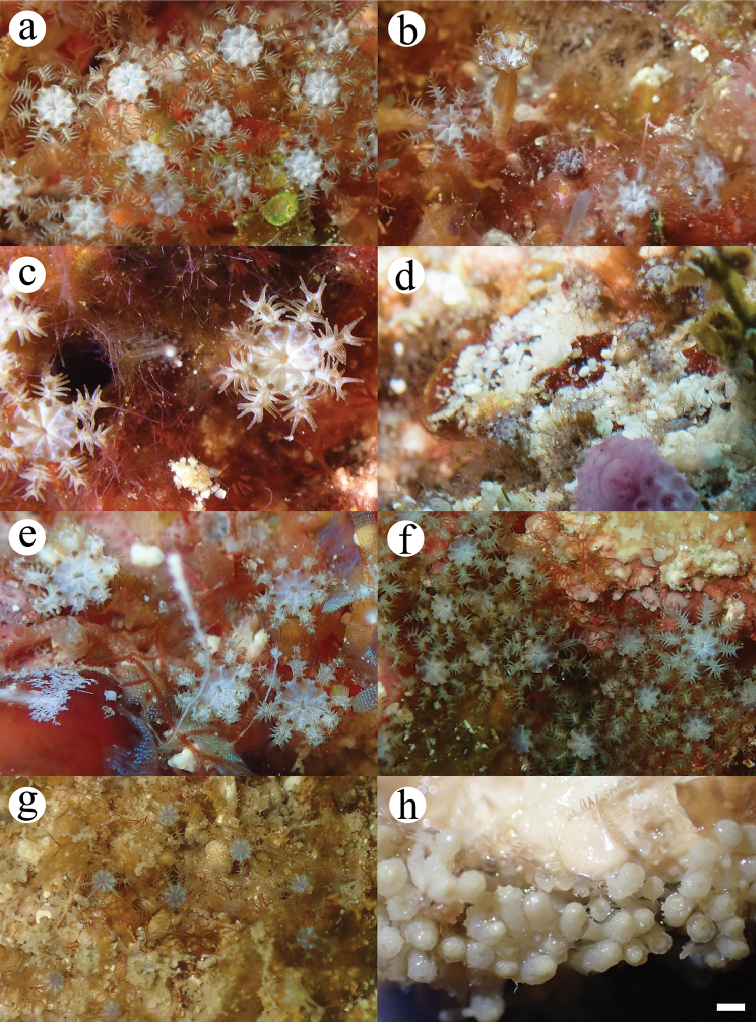
In situ photographs of examined *Hana* specimens from Okinawa, **a***Hanahanagasa*, holotype, OKA170711-15 and **b***Hanahanagasa*, paratype, OKA170711-06; Palau **c***Hanahanataba* holotype, ROR171225-01 and **d***Hanahanataba*, paratype, ROR171226-03; Dongsha **e***Hanahanataba*, paratype, DSX180320-1-01 and **f***Hanahanataba*, paratype, DSX180324-3-15 **g** specimen BKI180320-2-10, an arulid photographed in Tunku Abdul Rahman Park, Sabah, Malaysia **h***Hanahanagasa*, holotype, OKA170711-15, colony preserved in ethanol. Scale bar: 1 mm.

The oral disk expands into a broad circular membrane by fusion of the proximal regions of the adjacent tentacles. The margin of the oral membrane has eight broad lobes, with eight deep furrows, which run from the intertentacular margin to the mouth of the polyp, giving a plump appearance (Figure 2a). The distal two-thirds of the tentacles extend from fused margins of the oral membrane. Tentacles are long and thin, with 10 pairs of widely spaced pinnules, which are arranged in a single row on either side of the rachis.

**Figure 3.**
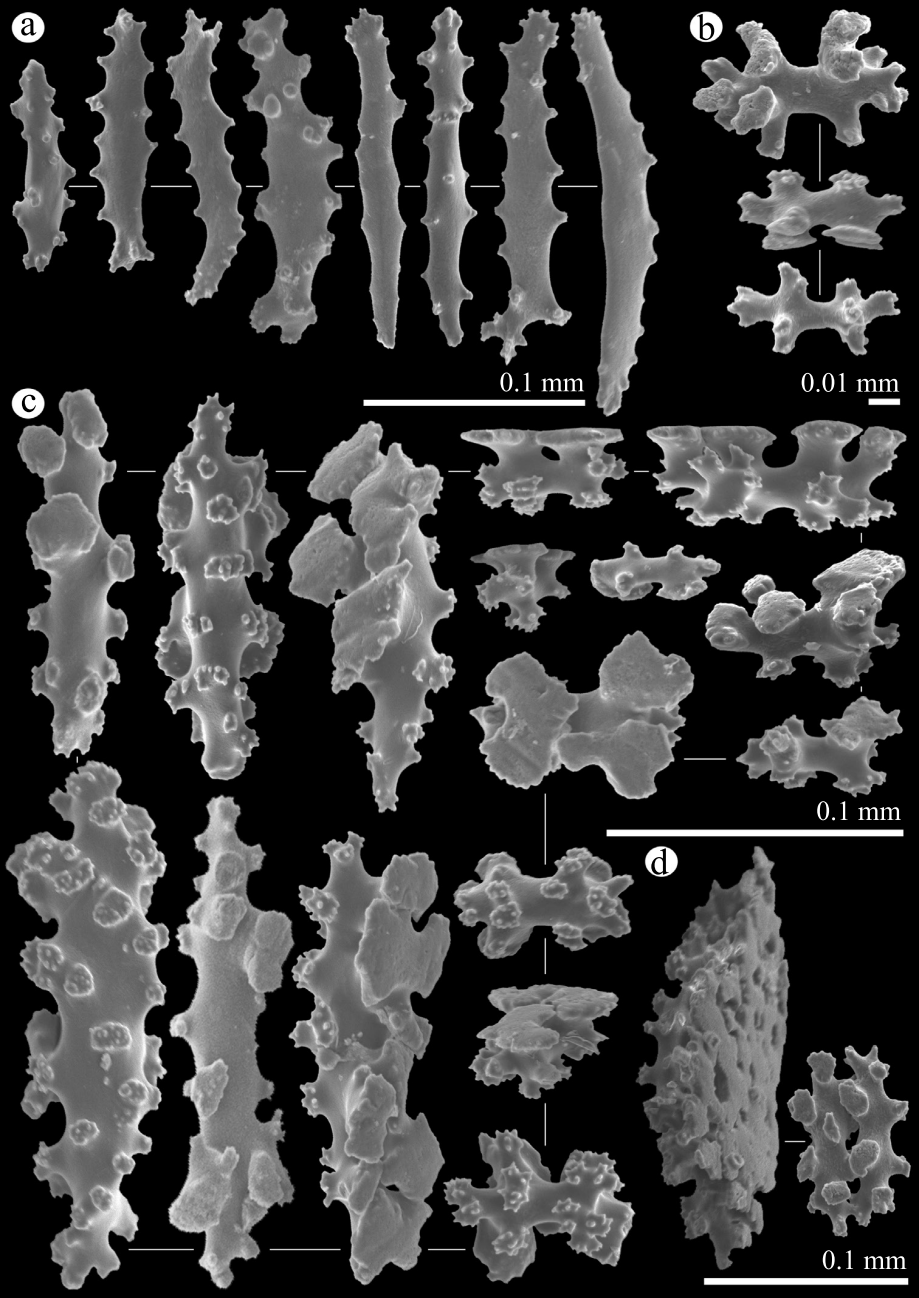
*Hanahanagasa*, holotype, OKA170711-15: **a** anthocodial rods **b** 6-radiates of calyx **c** table-radiates of calyx **d** pieces of fused table-radiates of stolon.

Anthocodial sclerites are small rods with simple tubercles around margins 0.10–0.18 mm long (Figure 3a). Calyx containing small 6-radiates 0.05–0.06 mm long (Figure 3b) and table-radiates ranging 0.03–0.17 mm, giving the largest table-radiates a spindle- and sometimes club-like appearance (Figure 3c). Stolons with fused table-radiates form a flat network (Figure 3d).

The oral disk is white and the tentacles are brown in life (Figure 2a), yellowish-white in ethanol (Figure 2h). Zooxanthellate.

####### Morphological variation.

Paratypes are colonies consisting of 50–100 polyps, growing on hard substrates and sponges. Colonies show variations in number of pinnules, having 8–10 pairs lining either side of the rachis.

####### Distribution.

Northwest coast of Okinawa Island and southeast coast of Iheya Island in the East China Sea.

####### Remarks.

*Arula* and *Hana* are the only two genera within the family Arulidae. *Arulapetunia* and *H.hanagasa* have very similar polyp morphologies with only a clear difference in polyp colour. Oral disk and tentacles of *A.petunia* are blue in life and white and brown in *H.hanagasa*, respectively. This would suggest assignment to the same genus, however, the combination of differences in genetic data and sclerite morphology indicate that they should be separate from each other at the generic level. The possibility that there are similar species or previous descriptions and reports on arulid species has previously been discussed (McFadden and Ofwegen 2012) and so far, no reports have been made on possible congeners.

####### Etymology.

From the Japanese language ‘hanagasa’ (花笠), the traditional Okinawan ceremonial dance headpiece worn by female performers; denoting the shape of the polyps, which resembles the flower headpiece.

###### Hana hanataba sp. n.

Taxon classificationAnimaliaAlcyonaceaArulidae

http://zoobank.org/578DEA2C-046E-498C-B0FE-0777A213209D

Figures 2c , 4

####### Material examined.

Holotype: ROR171225-01, Blue Corner, Ngemelis Island, Palau (7°8.400'N, 134°13.200'E), 23 m depth, coll. YW Lau, 25 July 2017 (MH845550; MH844386). Paratype 1: ROR171226-03, Peleliu PICRC monitoring site, Peleliu, Palau (7°0.400'N, 134°13.060'E), 28 m depth, coll. GY Soong, 26 December 2017 (MH845551; MH845543; MH844387). Paratype 2: DXS180420-1-01, North spur & grooves, Dongsha Atoll, Taiwan (20°46.291'N, 116°46.057'E), 7 m depth, coll. YW Lau, 20 April 2018 (MH845548). Paratype 3: DSX180424-3-15, North, Dongsha Atoll, Taiwan (20°46.677'N, 116°50.090'E), 8 m depth, coll. JD Reimer, 24 April 2018 (MH845549; MH845542; MH844385).

####### Description.

The colony consists of small polyps (~30) growing on rock. Polyps are spaced apart irregularly (0.5–2.5 mm), connected by stolons that are 0.5 mm in diameter and flat thin ribbon-like in cross-section. Polyps have anthocodia retracted into calyces of 2.5–3.0 mm tall and up to 1.0 mm diameter at the widest point; calyces are slightly club-shaped or barrel shaped, wider near the distal end than at the proximal point of attachment to the stolon.

The oral disk expands into a broad circular membrane by fusion of the proximal regions of the adjacent tentacles (Figure 2c). The margin of the oral membrane has eight broad lobes, with eight deep furrows, which run from the intertentacular margin to the mouth of the polyp, giving a plump appearance. The distal two-thirds of the tentacles extend from fused margins of the oral membrane. Tentacles are long and thin, with eight pairs of widely spaced pinnules, which are arranged in a single row on either side of the rachis.

**Figure 4.**
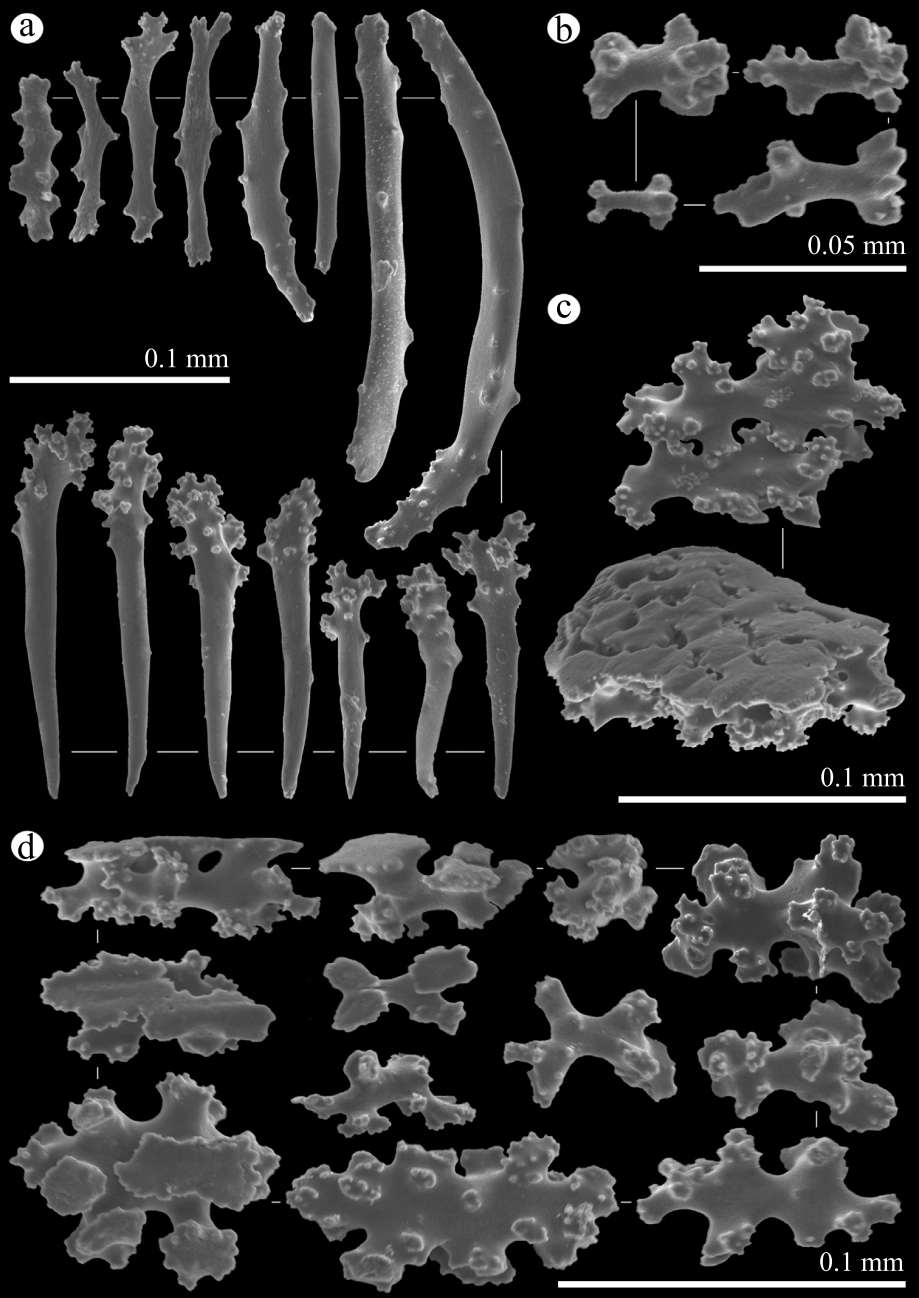
*Hanahanataba*, holotype, ROR171225-01: **a** anthocodial rods **b** capstans of calyx **c** pieces of fused table-radiates of stolon **d** table-radiates of calyx.

Anthocodial sclerites are rods with sparse simple tubercles around margins 0.07–0.24 mm long and rods ornamented with clustered tubercles on one end, giving it a club-shaped appearance, size 0.10–0.18 mm (Figure 4a). Calyx containing small capstans 0.02–0.05 mm long (Figure 4b) and table-radiates ranging 0.03–0.09 mm (Figure 4d). Sclerites of stolon are fused table-radiates forming a flat network (Figure 4c).

The oral disk and tentacles are white in life with brown in the proximal part of tentacle (Figure 2c), yellowish-white in ethanol. Zooxanthellate.

####### Morphological variation.

Paratypes consist of colonies with 30–100 polyps, growing on hard substrates. Colonies show variations in the tentacles, sometimes having ten pairs of pinnules.

####### Distribution.

The south-east of Palau in the Philippine Sea and the north to north-east reef of Dongsha Atoll, Taiwan in the South China Sea.

####### Remarks.

*Hanahanagasa* and *Hanahanataba* have very similar polyp morphology, with minor colour differences, which could be due to differing abundances of zooxanthellae. Genetic data and sclerite morphology indicate that *H.hanagasa* and *H.hanataba* should be separated from each other at the species level. Sclerites found in *H.hanataba* are different from those in *H.hanagasa* in the presence of ornamented rods, which are lacking in *H.hanagasa*. It is noteworthy that both *H.hanagasa* and *H.hanataba* were found in environments with the presence of a comparatively strong current.

####### Etymology.

From the Japanese language ‘hanataba’ (花束), meaning bouquet; denoting the multitude of polyps resembling arranged flowers.

#### Molecular phylogenetic analyses

**Figure 5.**
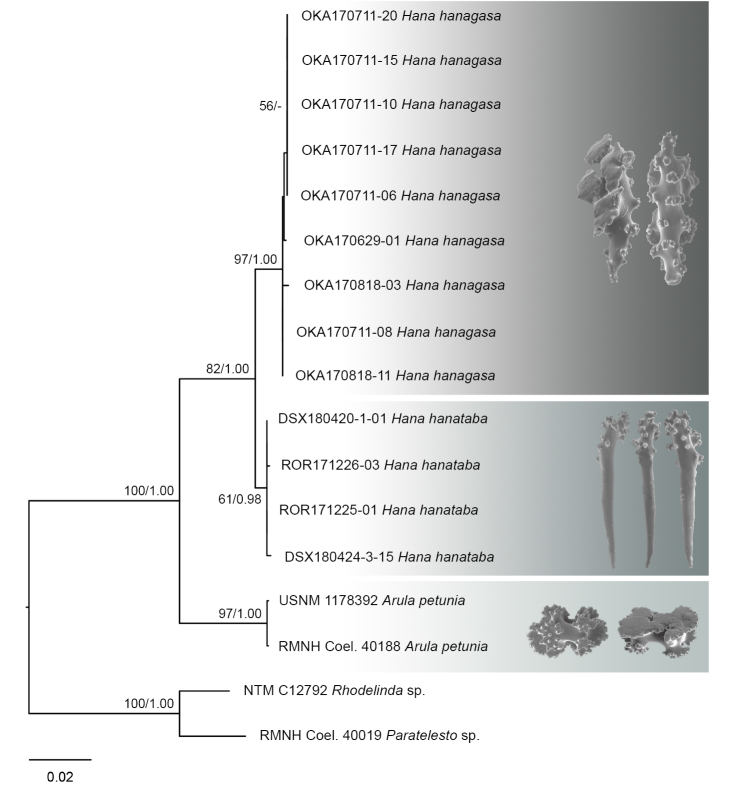
Phylogenetic reconstruction for arulid specimens from Okinawa Island (OKA), Palau (ROR), Dongsha Atoll (DSX), arulid reference taxa (*Arulapetunia*) and outgroup sister taxa (*Paratelesto* sp. and *Rhodelinda* sp.) using the combined COI+mtMutS+28S rDNA dataset. The best maximum likelihood tree is shown, with values at branches representing bootstrap probabilities (>50%) and posterior probabilities from the Bayesian inference analysis (>0.50), respectively. Sclerites unique to *Hanahanagasa* and *Hanahanataba* are shown and typical table radiates found in the family Arulidae are shown for the genus *Arula*.

This study has added 24 sequences to the reference database, representing two species for which no barcodes have been sequenced before. The phylograms resulting from the ML analyses of the separate markers were highly congruent with those from the analysis of the combined markers (Figure 5). ML and Bayesian analyses of the combined dataset yielded almost identical tree topologies.

##### *Hanahanagasa* and *Hanahanataba* from the north-western Pacific

Sequences of *H.hanagasa*, gen. n., sp. n., from Okinawa Island and *H.hanataba*, gen. n., sp. n., from Palau and Dongsha Atoll, Taiwan, grouped together in a well-supported clade within the Arulidae. The sequences formed a separate clade from sequences of *Arulapetunia* (Figure 5). Molecular phylogenetic analyses support the distinctiveness of the genera *Hana* and *Arula*. The genetic distances (uncorrected *p*, expressed as percentage) between *Arula* and *Hana* taxa were 3.54% and 5.36% for COI and mtMutS, respectively [Suppl. material 2]. This is on the far upper end of the range typical of differences among congeneric octocoral species (McFadden et al. 2011). The major ramification and distances indicate a separation of *H.hanagasa*, gen. n., sp. n., and *H.hanataba*, gen. n., sp. n., from *Arulapetunia* at the generic level. Morphological features support this division; comparing sclerite characteristics between *Arula* and *Hana* specimens, there are several differences that stand out. The main morphological difference are the table-radiates of the stolon in genus *Hana*, which are fused together into a flattened network. The table-radiates of *Arulapetunia* are of a smaller size range and lack two shapes of table-radiates that are seen in *Hanahanagasa* specimens, somewhat spindle and club shaped table-radiates. Additionally, there are differences in anthocodial rods; differing in size range, larger in *Hana* specimens. Additionally *Arula* specimens lack ornamented rods that are seen in *Hanahanataba* specimens (Figs 3–5).

The separation of *H.hanagasa* and *H.hanataba* was confirmed by molecular analyses and through the investigation of the sclerites. The genetic distances (uncorrected *p*, expressed as percentage) within the genus *Hana* between *H.hanagasa* and *H.hanataba* were 0.8% and 0.67% for COI and mtMutS, respectively [Suppl. material 2]. These percentages are far above margins typical of differences among intraspecific octocorals (McFadden et al. 2011). Specimens of the two species do not differ much in polyp morphology (Figure 2); however, investigation of the sclerites demonstrated differences between characters of sclerite morphology (Figs 3–4). Next to anthocodial rods with sparse tubercles arranged on the margins, a second type of rod was seen in *H.hanataba*; rods that are ornamented with lumps of tubercles on one end, giving the rods a club-like shape. Additionally, the table-radiates of the calyx seen in *H.hanataba* are of a smaller size range than of those in *H.hanagasa*, being much more similar to the table-radiates seen in *Arulapetunia*. These morphological characters unique to *H.hanagasa* and *H.hanataba* are projected on the phylogenetic tree (Figure 5).

##### Accession numbers

MH844382

MH844383

MH844384

MH844385

MH844386

MH844387

MH845552

MH845553

MH845554

MH845555

MH845556

MH845557

MH845558

MH845559

MH845548

MH845549

MH845550

MH845551

MH845544

MH845545

MH845546

MH845547

MH845542

MH845543

## Discussion

The new species *H.hanagasa* and *H.hanataba* bring the total number of species within the Arulidae to three and represent the first confirmed records of arulids for the north-western Pacific. Arulids so far have been recorded only in South Africa with informal reports of other possible congeners occurring in Sabah, Malaysia (Figure 2g) and a photographic record of a congener in Bali, Indonesia (McFadden and Ofwegen 2012). Even though it is still unknown what genus the photographed specimens from Bali and Sabah belong to, the current study expands the zoogeographical distribution of members of family Arulidae from the type locality (South Africa) to the north-western Pacific.

Even though all new arulid specimens were amplified for four markers, here we used only three of the gene regions, 28S rDNA, COI and mtMutS, in the analyses. ND6 was excluded from the analyses, as the outgroup sequences lacked available ND6 sequences. There were no differences in results when including or excluding this region when performing analyses with concatenated datasets. However, utilising four region sequences resulted in better resolution. Therefore, for future analyses, it is recommended to include ND6 to obtain better resolution.

It has been made clear in previous studies that the subordinal group Stolonifera is polyphyletic (McFadden et al. 2006, McFadden and Ofwegen 2012, Conti-Jerpe and Freshwater 2017). There is still much work needed, with families in need of formal description in order to reflect adequately the phylogenetic distribution of stoloniferous genera. The addition of the genus *Hana* and its two species to the recently erected family Arulidae is a small step in the process to fully interpret the morphological and molecular distinctions amongst clades of Stolonifera and ultimately the Octocorallia. It is clear that many new records of Stolonifera still await discovery and that this group still has morphological surprises, such as previously discovered pinnuleless tentacles, sclerite-free clavulariids, and new sclerite types (Bayer et al. 1983, Williams 2000, Alderslade and McFadden 2007, 2011, McFadden and Ofwegen 2012).

## Supplementary Material

XML Treatment for Arulidae

XML Treatment for Hana

XML Treatment for Hana hanagasa

XML Treatment for Hana hanataba

## Supplementary materials

Supplementary material 1 Supplemental figures 1–3 Data type: multimedia Explanation note: Figure 1: Maximum likelihood phylogenetic reconstruction of gene region 28S rDNA of arulid specimen from Okinawa (OKA), Palau (ROR), Dongsha Atoll (DSX), reference taxa *Arulapetunia* and outgroup sister taxa (*Paratelesto* sp. and *Rhodelinda* sp.). Figure 2: Maximum likelihood phylogenetic reconstruction of gene region COI of arulid specimen from Okinawa (OKA), Palau (ROR), Dongsha Atoll (DSX), reference taxa *Arulapetunia* and outgroup sister taxa (*Paratelesto* sp. and *Rhodelinda* sp.). Figure 3: Maximum likelihood phylogenetic reconstruction of gene region mtMutS of arulid specimen from Okinawa (OKA), Palau (ROR), Dongsha Atoll (DSX), reference taxa *Arulapetunia* and outgroup sister taxa (*Paratelesto* sp.). This is an open access article distributed under the terms of the Creative Commons Attribution License (CC BY 4.0), which permits unrestricted use, distribution, and reproduction in any medium, provided the original author and source are credited.Yee Wah Lau, Frank Robert Stokvis, Leendert Pieter van Ofwegen, James Davis Reimer

Supplementary material 2 Supplemental tables 1, 2 Data type: measurement Explanation note: Table 1. Numbers of base differences per site from averaging over all sequence pairs between groups (d) shown for gene regions COI and mtMutS (uncorrected *p*, expressed as percentage). Table 2. Estimates of average evolutionary divergence over sequence pairs within groups for gene regions COI and mtMutS. This is an open access article distributed under the terms of the Creative Commons Attribution License (CC BY 4.0), which permits unrestricted use, distribution, and reproduction in any medium, provided the original author and source are credited.Yee Wah Lau, Frank Robert Stokvis, Leendert Pieter van Ofwegen, James Davis Reimer

## References

[B1] Aberer AJ Krompass D Stamatakis A (2013) Pruning rogue taxa improves phylogenetic accuracy: an efficient algorithm and webservice. Systematic Biology 62: 162–166. 10.1093/sysbio/sys078 22962004 PMC3526802

[B2] Aberer AJ Kobert K Stamatakis A (2014) ExaBayes: massively parallel Bayesian tree inference for the whole-genome era. Molecular Biology and Evolution 31: 2553–2556. 10.1093/molbev/msu236 25135941 PMC4166930

[B3] Alderslade P McFadden CS (2007) Pinnule-less polyps: a new genus and new species of Indo-Pacific Clavulariidae and validation of the soft coral genus *Acrossota* and the family Acrossotidae (Coelenterata: Octocorallia). Zootaxa 1400: 27–44. 10.11646/zootaxa.1400.2

[B4] Alderslade P McFadden CS (2011) A new sclerite-free genus and species of Clavulariidae (Coelenterata: Octocorallia). Zootaxa 3104: 64–68. http://www.mapress.com/zootaxa/2011/f/z03104p068f.pdf

[B5] Bayer FM Grasshoff M Verseveldt J (1983) Illustrated trilingual glossary of morphological and anatomical terms applied to octocorallia. EJ Brill/Dr W Backhuys, Leiden, 75 pp http://www.cap-recifal.com/ccs_files/articles/octo1_denisio/guide-bayer.pdf

[B6] Conti-Jerpe IE Freshwater DW (2017) *Hederacaerulescens* (Alcyonacea: Alcyoniidae), a new genus and species of soft coral from the temperate North Atlantic: invasive in its known range? Invertebrate Systematics 31: 723–33. 10.1071/IS16069

[B7] Churashima Foundation, Biological Institute on Kuroshio (2016) Soft corals of Okinawa – catalogue of the collection of the Churashima Research Center. [In Japanese]

[B8] Cordeiro R Ofwegen LP Williams G (2018) World List of Octocorallia Clavulariidae Hickson, 1894. http://www.marinespecies.org/aphia.php?p=taxdetails&id=125270

[B9] Daly M Brugler MR Cartwright P Collins AG Dawson MN Fautin DG France SC McFadden CS Opresko DM Rodriguez E Romano SL Stake JL (2007) The phylum Cnidaria: A review of phylogenetic patterns and diversity 300 years after Linnaeus. Zootaxa 1668: 127–182. http://hdl.handle.net/1808/13641

[B10] Doorenweerd C (2016) PhylOStack: a phylogenetic analyses pipeline in Linux Ubuntu 14.04 using OpenStack cloud computing. https://github.com/naturalis/openstack-docs/wiki

[B11] Fabricius K Alderslade P (2001) Soft corals and sea fans: A comprehensive guide to the shallow-water genera of the central-west Pacific, the Indian Ocean and the Red Sea. Australian Institute of Marine Science, Townsville, 264 pp http://epubs.aims.gov.au/11068/5817

[B12] Foster PG (2004) Modeling compositional heterogeneity. Systematic Biology 53: 485–495. 10.1080/10635150490445779 15503675

[B13] France SC Hoover LL (2002) DNA sequences of the mitochondrial *COI* gene have low levels of divergence among deep-sea octocorals (Cnidaria: Anthozoa). Hydrobiologia 471: 149–155. https://link.springer.com/article/10.1023/A:1016517724749

[B14] Gaurav V Lohman DJ Meier R (2011) SequenceMatrix: concatenation software for the fast assembly of multi-gene datasets with character set and codon information. Cladistics 27: 171–180. 10.1111/j.1096-0031.2010.00329.x 34875773

[B15] Hall TA (1999) BioEdit: a user-friendly biological sequence alignment editor and analysis program for Windows 95/98/NT. Nucleic Acids Symposium Series 41: 95–98. http://jwbrown.mbio.ncsu.edu/JWB/papers/1999Hall1.pdf

[B16] Katoh K Standley DM (2013) MAFFT multiple sequence alignment software version 7: improvements in performance and usability. Molecular Biology and Evolution 30(4): 772–780. https//10.1093/molbev/mst010 23329690 PMC3603318

[B17] Kearse M Moir M Wilson A Stones-havas S Cheung M Sturrock S Buxton S Cooper A Markowitz S Duran C Thierer T Ashton B Meintjes P Drummond A (2012) Geneious Basic: An integrated and extendable desktop software platform for the organization and analysis of sequence data. Bioinformatics 28: 1647–1649. 10.1093/bioinformatics/bts199 22543367 PMC3371832

[B18] Kumar S Stecher G Tamura K (2016) MEGA7: Molecular Evolutionary Genetics Analysis Version 7.0 for bigger datasets. Molecular Biology and Evolution 33(7): 1870–1874. 10.1093/molbev/msw054 27004904 PMC8210823

[B19] Larsson A (2014) AliView: a fast and lightweight alignment viewer and editor for large datasets. Bioinformatics 30: 3276–3278. 10.1093/bioinformatics/btu531 25095880 PMC4221126

[B20] McFadden CS France SC Sánchez JA Alderslade P (2006) A molecular phylogenetic analysis of the Octocorallia (Coelenterata: Anthozoa) based on mitochondrial protein-coding sequences. Molecular Phylogenetics and Evolution 41: 513–527. 10.1016/j.ympev.2006.06.010 16876445

[B21] McFadden CS Benayahu Y Pante E Thoma JN Nevarez PA France SC (2011) Limitations of mitochondrial gene barcoding in Octocorallia, Molecular Ecology Resources 11: 19–31. 10.1111/j.1755-0998.2010.02875.x 21429097

[B22] McFadden CS van Ofwegen LP (2012) Stoloniferous octocorals (Anthozoa, Octocorallia) from South Africa, with descriptions of a new family of Alcyonacea, a new genus of Clavulariidae, and a new species of *Cornularia* (Cornulariidae). Invertebrate Systematics 26: 331–356. 10.1071/IS12035

[B23] McFadden CS van Ofwegen LP (2013) A second, cryptic species of the soft coral genus *Incrustatus* (Anthozoa: Octocorallia: Clavulariidae) from Tierra del Fuego, Argentina, revealed by DNA barcoding. Helgoland Marine Research 67: 137–147. 10.1007/s10152-012-0310-7

[B24] van Ofwegen LP Häussermann V Försterra G (2006) A new genus of soft coral (Octocorallia: Alcyonacea: Clavulariidae) from Chile. Zootaxa 1219: 47–57. http://citeseerx.ist.psu.edu/viewdoc/download?doi=10.1.1.439.2939&rep=rep1&type=pdf

[B25] Rambout A (2014) FigTree. http://tree.bio.ed.ac.uk/software/figtree/

[B26] Rambaut A Suchard MA Xie D Drummond AJ (2014) Tracer v1.6. http://beast.bio.ed.ac.uk/Tracer

[B27] Sánchez JA McFadden CS France SC Lasker HR (2003) Molecular phylogenetic analyses of shallow-water Caribbean octocorals. Marine Biology 142: 975–987. 10.1007/s00227-003-1018-7

[B28] Stamatakis A (2014) RAxML version 8: a tool for phylogenetic analyses and post-analysis of large phylogenies. Bioinformatics 30(9): 1312–1313. 10.1093/bioinformatics/btu033 24451623 PMC3998144

[B29] Weinberg S (2017) Découvrir la vie sous-marine, Mer Rouge, ocean Indien, ocean Pacifique. Guide d’identification, 1001 espèces de fauna et flore. Tome 1: 1–261.

[B30] Williams GC (2000) A new genus and species of stoloniferous octocoral (Anthozoa: Clavulariidae) from the Pacific coast of North America. Zoologische Mededelingen Leiden 73: 333–344. http://citeseerx.ist.psu.edu/viewdoc/download?doi=10.1.1.844.3643&rep=rep1&type=pdf

[B31] Williams GC (2013) New taxa and revisionary systematics of alcyonacean octocorals from the Pacific coast of North America (Cnidaria, Anthozoa). Zookeys 283: 15–42. 10.3897/zookeys.283.4803 PMC3677362 23794840

