# Corrigenda: *Hanah*, a replacement name for *Hana* Lau, Stokvis, Ofwegen & Reimer, 2018 (preoccupied name)

**DOI:** 10.3897/zookeys.918.51123

**Published:** 2020-03-12

**Authors:** Yee Wah Lau, Frank R. Stokvis, Leen van Ofwegen, James D. Reimer

**Affiliations:** 1 University of the Ryukyus, Nishihara, Okinawa, Japan University of the Ryukyus Nishihara Japan; 2 Naturalis Biodiversity Center, Leiden, Netherlands Naturalis Biodiversity Center Leiden Netherlands

**Keywords:** Arulidae, *
Hana
*, homonymy, Octocorallia, replacement name, Stolonifera

## Abstract

This is an addendum to the availability of the generic name *Hana* Lau, Stokvis, Ofwegen & Reimer, 2018 within the octocoral family Arulidae. Here, the replacement name *Hanah* is proposed, as *Hana* Lau, Stokvis, Ofwegen & Reimer, 2018 is a junior homonym to senior homonym *Hana* Kukalova-Peck, 1975, a genus within the family Hanidae of the Palaeozoic insect order Megasecoptera.

## Class Anthozoa

### Subclass Octocorallia

**Order Alcyonacea**

**Suborder Stolonifera**

**Family Arulidae**

**Genus *Hanah* nom. nov.**

*Hana* Lau, Stokvis, Ofwegen & Reimer, 2018. ZooKeys 790: 1–19. (Anthozoa: Octocorallia: Alcyonacea: Stolonifera: Arulidae). Preoccupied by *Hana* Kukalova-Peck, 1975. Psyche 82: 1–19. (Insecta: Pterygota: Palaeoptera: Palaeodictyopteroidea: Megasecoptera: Hanidae).

Following the publication of Lau et al. (2018), it has been brought to our attention that the genus name *Hana* is a preoccupied genus name within the Palaeozoic insect order Megasecoptera (Kukalova-Peck 1975) as documented in Nomenclator Zoologicus (1975: 205). The name *Hana* Lau, Stokvis, Ofwegen & Reimer, 2018 is thus invalid under the laws of homonymy, being a junior homonym of *Hana* Kukalova-Peck, 1975.

In accordance with article 60 of the International Code of Zoological Nomenclature, fourth edition (1999), the junior homonym *Hana* Lau, Stokvis, Ofwegen & Reimer, 2018 will be replaced by *Hanah* nom. nov.

According to this application, the following new combinations within *Hanah* (Lau, Stokvis, Ofwegen & Reimer, 2018) are *Hanah hanagasa* (Lau, Stokvis, Ofwegen & Reimer, 2018), comb. nov. and *Hanah hanataba* (Lau, Stokvis, Ofwegen & Reimer, 2018), comb. nov.
