# Supplementary material for: Stolonifera from shallow waters in the north-western Pacific: a description of a new genus and two new species within the Arulidae (Anthozoa, Octocorallia)
Source: Zookeys. 2018 Oct 15;(790):1–19. doi: 10.3897/zookeys.790.28875 (PMC6198026; doi:10.3897/zookeys.790.28875)
Supplement: Supplementary material 2 — Supplemental tables 1, 2 [file zookeys-790-001-s002.docx]

**Supplementary material 2**

Supplemental Table 2. Estimates of average evolutionary divergence over sequence pairs within groups for gene regions COI and mtMutS. The numbers of base differences per site from averaging over all sequence pairs within each group (d) are shown (uncorrected *p*, expressed as percentage). Standard error estimates (S.E.) are shown in the second column and were obtained by a bootstrap procedure (1000 replicates). Analyses involved 14 and 8 nucleotide sequences for COI and mtMutS, respectively. All positions containing gaps and missing data were eliminated. There were a total of 809 positions in the final dataset. Evolutionary analyses were conducted in MEGA7 (Kumar et al. 2016).

|  |  | COI |  | MSH |  |
| --- | --- | --- | --- | --- | --- |
| Within groups |  | d | S.E. | d | S.E. |
| species | *H. hanagasa* | 0.0000 | 0.0000 | 0.0000 | 0.0000 |
|  | *H. hanataba* | 0.0000 | 0.0000 | 0.0015 | 0.0014 |
| genus | *Hana* | 0.0042 | 0.0015 | 0.0037 | 0.0015 |
|  | *Arula* | 0.0000 | 0.0000 | 0.0000 | 0.0000 |
